# Characterization of genetically engineered mouse models carrying Col2a1-cre-induced deletions of Lrp5 and/or Lrp6

**DOI:** 10.1038/boneres.2015.42

**Published:** 2016-03-01

**Authors:** Cassie A Schumacher, Danese M Joiner, Kennen D Less, Melissa Oosterhouse Drewry, Bart O Williams

**Affiliations:** 1 Center for Cancer and Cell Biology, Program in Skeletal Disease and Tumor Microenvironment, Van Andel Research Institute, Grand Rapids MI 49503, USA

## Abstract

Mice carrying *Collagen2a1-cre*-mediated deletions of Lrp5 and/or Lrp6 were created and characterized. Mice lacking either gene alone were viable and fertile with normal knee morphology. Mice in which both Lrp5 and Lrp6 were conditionally ablated via *Collagen2a1*-cre-mediated deletion displayed severe defects in skeletal development during embryogenesis. In addition, adult mice carrying *Collagen2a1-cre*-mediated deletions of Lrp5 and/or Lrp6 displayed low bone mass suggesting that the Collagen2a1-cre transgene was active in cells that subsequently differentiated into osteoblasts. In both embryonic skeletal development and establishment of adult bone mass, Lrp5 and Lrp6 carry out redundant functions.

## Introduction

The Wnt/β-catenin signaling pathway affects cell migration, proliferation, and differentiation and is a key regulator of skeletal development.^1^ This pathway is initiated when a Wnt ligand engages a receptor complex that includes a member of the Frizzled family of seven-transmembrane receptors and either low-density lipoprotein-related receptor 5 (Lrp5) or Lrp6.^2^ This results in the phosphorylation of the carboxyl terminus of Lrp5 or Lrp6, creating a binding site for Axin. Axin is a component of a multiprotein complex that also includes the Adenomatous polyposis coli protein and the serine/threonine protein kinase glycogen synthase kinase 3 (GSK3). In the absence of Wnt, this complex facilitates the GSK3-dependent phosphorylation of β-catenin, targeting it for ubiquitin-dependent proteolysis. Binding of Axin to the phosphorylated carboxyl terminus of Lrp5/6 inhibits GSK3 activity toward β-catenin, resulting in stabilization of β-catenin in the cytoplasm. β-catenin is then available to enter the nucleus where it complexes with members of the LEF/TCF family of DNA-binding proteins and induces the activation of target gene promoters.

Numerous studies have indicated that alterations in Wnt/β-catenin levels can influence chondrocyte differentiation and/or function.^3–11^ For example, ectopic expression of β-catenin in chondrocyte lineage cells inhibits chondrocyte differentiation. Increased levels of canonical Wnt/β-catenin signaling may inhibit Sox9 expression and activity thus reducing chondrocyte differentiation.^12^ In addition, both increased and decreased β-catenin signaling has been observed in degenerative osteoarthritis (OA) models.^13–18^ Lrp5 mRNA and protein is increased in cells from human OA patients and Lrp5 polymorphisms analyzed as a group were correlated with human OA.^19–23^

During the last decade, we and others have utilized genetically engineered mouse models to gain insight into the roles of components of the Wnt pathway in skeletal development (reviewed in ref. 1). Germline deletion of Lrp6 leads to neonatal lethality associated with defects in the axial skeletal and limbs.^24^ Tissue-specific and heterozygous mutations that inactivate Lrp6 early in development produce skeletal phenotypes that are typically more severe than those in mice with mutations in Lrp5.^24–26^ Gaining insight into how Wnt/β-catenin signaling influences the development and function of normal cartilage will provide insight into utilizing Lrp5/6 as therapeutic targets for OA.^27,28^

In this study, we examined the effect of loss of Lrp5 and Lrp6 function in Collagen2a1 expressing cells and their descendants in both developing and adult mouse skeletons. We used the cre-lox system in which cre was driven by the Col2a1 promoter,^29^ which is expressed by chondrocytes and osteoblast progenitors and has been used in a variety of animal models to achieve tissue-specific gene expression in chondrocytes.^7^^,^^30^ We generated mice with single conditional knockout mutations in either *Lrp5* or *Lrp6* and mice with combined mutations in *Lrp5* and *Lrp6*.

## Materials and methods

### Mouse strains

*Lrp5*^*flox*^, *Lrp6*^*flox*^, *Col2a1-cre*, and *mT/mG* mice have been described previously.^29,31–33^ All experiments were done in compliance with the Guidelines for the Care and Use of Animals for Scientific Research.^34^ The Institutional Animal Care and Use Committee of the Van Andel Research Institute approved all experimental procedures.

### PCR-based genotyping

Genomic DNA was extracted from tail biopsies using an AutoGenprep 960 automated DNA isolation system (AutoGen Inc., Holliston, MA, USA). PCR-based strategies were then used to genotype the mice (details available upon request).^35^ For allele-specific PCR, genomic DNA was extracted from various tissue types of mice using DNeasy Blood and Tissue Kit (Qiagen, Hilden, Germany).

### Whole-Mount Skeletal Staining

Whole-mount skeletal staining of embryos was performed according to McLeod with modifications.^36^ Briefly, skin and viscera were removed and embryos were placed in 95% EtOH for 1–2 days. Embryos (E18.5) were then placed in acetone for 1 day and then stained with alizarin red (0.1% alizarin red S in 95% EtOH) and alcian blue (0.3% alcian blue 8GX in 70% EtOH) for 3 days at 37 °C. Embryos were then dipped in dH_2_O, cleared in 1% aqueous KOH until nearly all of the soft tissue was gone, cleared in 80% 1% KOH/20% glycerol, cleared in 50% 1% KOH/50% glycerol, cleared in 20% 1% KOH/80% glycerol, and finally transferred to 100% glycerol for storage.

### Histology

To investigate the effect of Lrp5/6 loss of function driven by the Col2 promoter during embryonic development and in adult bone and cartilage, limbs from E16.5, E17.5, and E18.5 embryos Lrp5/6 mutant and WT mice and knees from 6-month-old male and female *Col2-cre;Lrp5*^*Fl/Fl*^, *Col2-cre;Lrp6*^*Fl/Fl*^, *Col2-cre;Lrp5*^*Fl/+*^*;Lrp6*^*Fl/+*^, *Col2-cre;Lrp5*^*Fl/FL*^*;Lrp6*^*Fl/+*^, and *Col2-cre;Lrp5*^*Fl/+*^*;Lrp6*^*Fl/FL*^ mice and respective WT littermates were fixed in 10% neutral buffered formalin for 48 h and decalcified with Immunocal (Decal Chemical Corporation, Tallman, NY, USA) for 48 h. Samples were infiltrated with an alcohol series, cleared with xylene, and infiltrated with paraffin. Samples were embedded on edge into a paraffin mold using the Leica Embedding center, sectioned at 5 μm using a microtome, collected onto glass slides, deparaffinized, and hydrated in distilled water. Sagittal knee sections were stained with Safranin O (0.1% Safranin O in distilled water) and Fast Green (0.05% Fast Green FCF in distilled water), and counterstained with Hematoxylin. Sections from embryos were stained with pentachrome according to the Movat method. Sections were imaged with a Nikon Eclipse 55i microscope and Nikon Digital Sight camera (Nikon, Melville, NY, USA).

### DEXA

Mice were anesthetized via inhalation of 2% isoflurane (TW Medical Veterinary Supply) with oxygen (1.0 L·min^−1^) for 10 min prior to imaging and during the procedure (⩽5 min). The mice were placed on a specimen tray in a PIXImus II bone densitometer (GE Lunar) for analysis. Bone mineral density (BMD) was calculated by the PIXImus software based on the active bone area in the subcranial region within the total body image and specifically in the femur, humerus, and axial skeleton.

### Microcomputed tomography

Trabecular and cortical BMD and architecture were assessed after collecting samples at 6 months of age using standardized methods^37^ at the distal femoral metaphysis and femoral midshaft, respectively, using a desktop SkyScan 1172 microCT imaging system (SkyScan, Kontich, Germany). Scans were acquired using a 13.3-μm^3^ isotropic voxel size, with 130 CT slices evaluated at the distal femur and 230 CT slices at the femoral midshaft. For trabecular and cortical bone analyses, fixed thresholds of 85 and 113, respectively, were used to determine the mineralized bone fraction. These values were calculated by averaging together all individual threshold levels. Individual CT slices were reconstructed with SkyScan reconstruction software and data were analyzed with CTan(Comprehensive TeX Archive Network). The region of interest of trabecular bone was drawn manually a few voxels away from the endocortical surface. The SkyScan was calibrated daily to hydroxyapatite cores of known density.

## Results

### Generation of mice carrying Col2-cre-mediated deletions in Lrp5 and/or Lrp6

**Figure 1:**
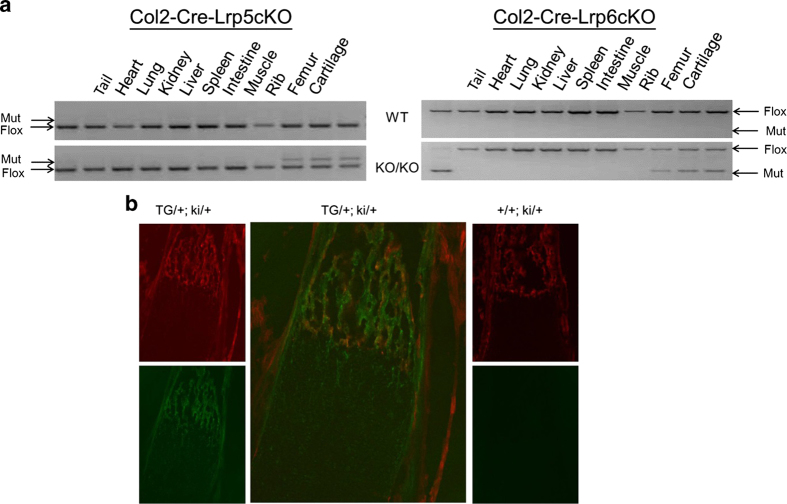
Evaluating *Col2-cre* activity and tissue-specific deletion of Lrp5 and Lrp6. (**a**) Allele-specific PCR was used to assess where cre-mediated activity resulted in an inactivated allele of Lrp5 or Lrp6 (mutant) and where the floxed allele remained intact (flox). The *Col2-cre*-Lrp5cKO mutant band was detected in the rib, femur, and cartilage, whereas the *Col2-cre*-Lrp6cKO mutant band was detected in the rib, femur, cartilage, and tail. (**b**) *Col2-cre* expressing mice were crossed to the mTmG reporter strain. The presence of Tomato protein (red fluoresence) marks cells that have not undergone cre-mediated recombination of the reporter gene, whereas the expression of GFP (green) is indicative of cells derived from *Col2-cre* expressing cells. The left panel demonstrates red (top) and green (top) fluoresence in sections from the femur of a *Col2-cre*;mtmG mouse (TG/+;ki/+), whereas the right panel shows the same staining for a femur from a control littermate (+/+;ki/+). The middle panel is a higher power view of a combination of the images in the left panels.

A detailed summary of the mouse strains and genetic backgrounds associated with this study is provided in Table 1. Mouse strains carrying floxed alleles of *Lrp5* and *Lrp6* have been previously described and have no deficit in Lrp5 or Lrp6 function in the absence of exposure to cre recombinase.^31^^,^^35^ These strains were crossed to mice expressing the cre recombinase under the control of the *Collagen2A1* promoter (*Col2-cre*).^29^ This process generated mice with several potential genotypes. *Col2-cre* expressing mice homozygous for either *Lrp5*^*flox*^ (*Col2-cre;Lrp5*^*Fl/Fl*^) or *Lrp6*^*flox*^ (*Col2-cre;Lrp6*^*Fl/Fl*^) were viable and fertile. In addition, mice carrying several different combinations of *Col2-cre*-induced in Lrp5 and Lrp6 were also viable. Mice heterozygous for both *Lrp5*^*flox*^ and *Lrp6*^*flox*^ (*Col2-cre;Lrp5*^*Fl/+*^*;Lrp6*^*Fl/+*)^ or heterozygous for one such allele and homozygous for the other (*Col2-cre;Lrp5*^*Fl/FL*^*;Lrp6*^*Fl/+*^ or *Col2-cre;Lrp5*^*Fl/+*^*;Lrp6*^*Fl/FL*^ were all viable and fertile. In contrast, mice homozygous for Col2-mediated deletion of both Lrp5 and Lrp6 (*Col2-cre;Lrp5*^*Fl/FL*^*;Lrp6*^*Fl/FL*^) died *in utero* or shortly after birth. Mice lacking the *Col2-cre* transgene are considered WT and used as controls for these studies as the Lrp5 and Lrp6 loci function normally in the absence of cre (Figure 1). A summary of the nomenclature used in the text and in the figures for the genotypic cohorts of mice in this study is provided in Table 2.

### Assessment of Col2-cre activity

To confirm the pattern of *Col2-cre* activity, allele-specific PCR was performed. In *Col2-cre;Lrp5*^*Fl/FL*^ mice, PCR products specifically derived from alleles that had undergone cre-mediated recombination were detected in the ribs, femur, and cartilage. In *Col2-cre;Lrp6*^*Fl/FL*^ mice, mutant bands were detected in the tail, ribs, femur, and cartilage (Figure 1a). To further confirm activity, we crossed *Col2-cre* transgenic mice to a strain carrying the mT/mG cre reporter.^33^ In the mT/mG mice, loxP sites are present around the tdTomato (mT) expression cassette. In the absence of Cre-mediated recombination, the tomato protein is expressed in all tissues which, as a result, display red fluorescence. Upon exposure to cre, recombination excises the mT expression cassette, allowing expression of green fluorescent protein (GFP). GFP expression, indicative of Cre recombination, was detected in hypertrophic chondrocytes and both trabecular and cortical bone regions of *Col2-cre*-expressing mice (Figure 1b).

### Col2-cre;Lrp5^Fl/FL^;Lrp6^Fl/FL^ mice have skeletal abnormalities during development

**Figure 2:**
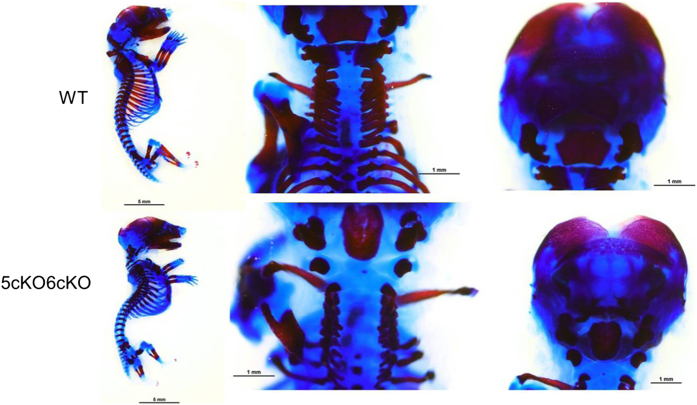
*Col2-cre;Lrp5*^*Fl/FL*^*;Lrp6*^*Fl/FL*^ (5cKO6cKO) embryos have significant defects in mineralization. E18.5 alizarin red and alcian blue stains of the entire skeleton (sagittal view) (left panels), top view of the spine and ribs (middle panel); and back of the cranium (right panels).

**Figure 3:**
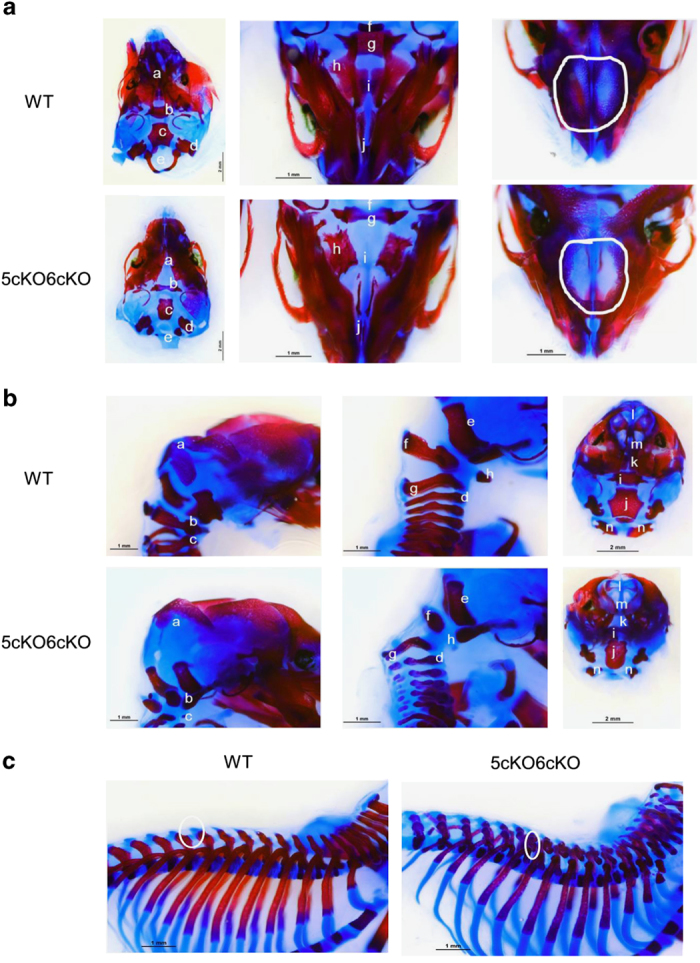
Higher magnification views of *Col2-cre*;Lrp5^Fl/FL^;Lrp6^Fl/FL^ (5cKO;6cKO) embryo mineralization defects. (**a**) E18.5 alizarin red and alcian blue stain of a top view of the skull with the supraoccipital bone removed; view from the underside of the skull; and top view of the skull looking down at the mouse nasal cavity. (**b**) E18.5 alizarin red and alcian blue stains of the skull and top cervical vertebra; base of the skull and cervical vertebra; and posterior view of the skull with the supraoccipital bone removed. Specific areas are noted in **a** and **b** by the following notations: a. Supraoccipital bone; b. Dorsal arch of the first cervical vertebrae (C1); c. C2 vertebra; d. Abnormal vertebral structure; e. Exocipital bone; f. Dorsal arch; g. C2 vertebrae; h. Cartilage primordium of body of hyoid bone. i. Basisphenoid bone; j. Basioccipital bone; k. Palatine bone; l. nasal bone; m. Putative extension of nasal bone; n. Dorsal arch of C1 vertebrae. (**c**) Representative example of alizarin red and alcian blue stains of the spine and ribs from a 5cKO6cKO E18.5 embryo and a WT littermate. WT, wild type.

Once it was apparent that *Col2-cre;Lrp5*^*Fl/FL*^*;Lrp6*^*Fl/FL*^ mice did not survive beyond birth, we set up timed matings and collected embryos at e18.5. To assess differences in skeletal development, whole mount staining of embryos with Alizarin Red and Alcian Blue was performed. We did not observe any gross differences between control embryos and mice homozygous for conditional deletion of either Lrp5 (*Col2-cre;Lrp5*^*Fl/FL*^) or Lrp6 *Col2-cre;Lrp6*^*Fl/FL*^; data not shown). In contrast, gross differences in skeletal morphology were observed between *Col2-cre;Lrp5*^*Fl/FL*^*;Lrp6*^*Fl/FL*^ and WT littermates at this stage (Figure 2). In *Col2-cre;Lrp5*^*Fl/FL*^*;Lrp6*^*Fl/FL*^ embryos, the supraoccipital bone was not mineralized; and the dorsal arches of the C1 vertebrae and C2 vertebrae were underdeveloped (Figure 3a and 3b). These mutant embryos had smaller exoccipial bones, little cartilage ossification in the primordium body of the hyoid bone, and a gap within vertebrae (Figure 3a and 3b). A lack of bone growth and fusion was observed in the palate and reduced membranous ossification of the nasal bone was also observed in *Col2-cre;Lrp5*^*Fl/FL*^*;Lrp6*^*Fl/FL*^ embryos. A lack of nasal bone ossification and growth was apparent in addition to limited growth and ossification of the palatine bone, and underdevelopment and irregular shape of basisphenoid bone (Figure 3a and b). There were smaller projections of the vertebrae and reduced mineralization within the spine and ribs (Figure 3c) of *Col2-cre;Lrp5*^*Fl/FL*^*;Lrp6*^*Fl/FL*^ embryos.

### Col2-cre;Lrp5^Fl/FL^;Lrp6^Fl/FL^ mice have altered cartilaginous zones during development

**Figure 4:**
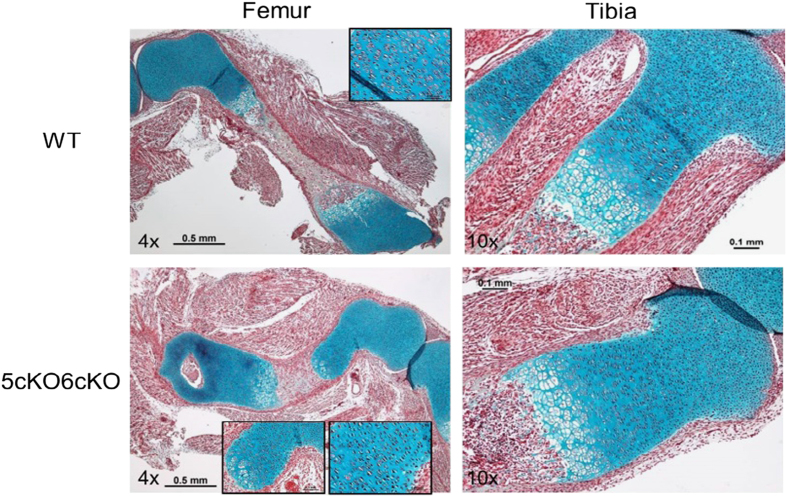
*Col2-cre;Lrp5*^*Fl/FL*^*;Lrp6*^*Fl/FL*^ (5cKO;6cKO) mice have altered cartilaginous zones during development. Cartilage (blue) in pentachrome stained femur (left panels) and tibia (right panels) sections from E18.5 5cKO6cKO (bottom panels) and WT littermates (top panels). Boxed regions are shown at higher magnification. WT, wild type.

Limbs from E18.5 *Col2-cre;Lrp5*^*Fl/FL*^*;Lrp6*^*Fl/FL*^ embryos were bowed with the hypertrophic zone extending further from the growth plate. This was associated with increased alcian blue staining (Figure 4). This phenotype is similar to that seen in *Dermo1-cre;Lrp5*^*Fl/FL*^*;Lrp6*^*Fl/FL*^ embryos.^31^

### Adult *Col2-cre;Lrp5*^*Fl/FL*^ or *Col2-cre;Lrp6*^*Fl/FL*^ mice and mice with combinatorial deletions of *Lrp5* and *Lrp6* have low bone mass

**Figure 5:**
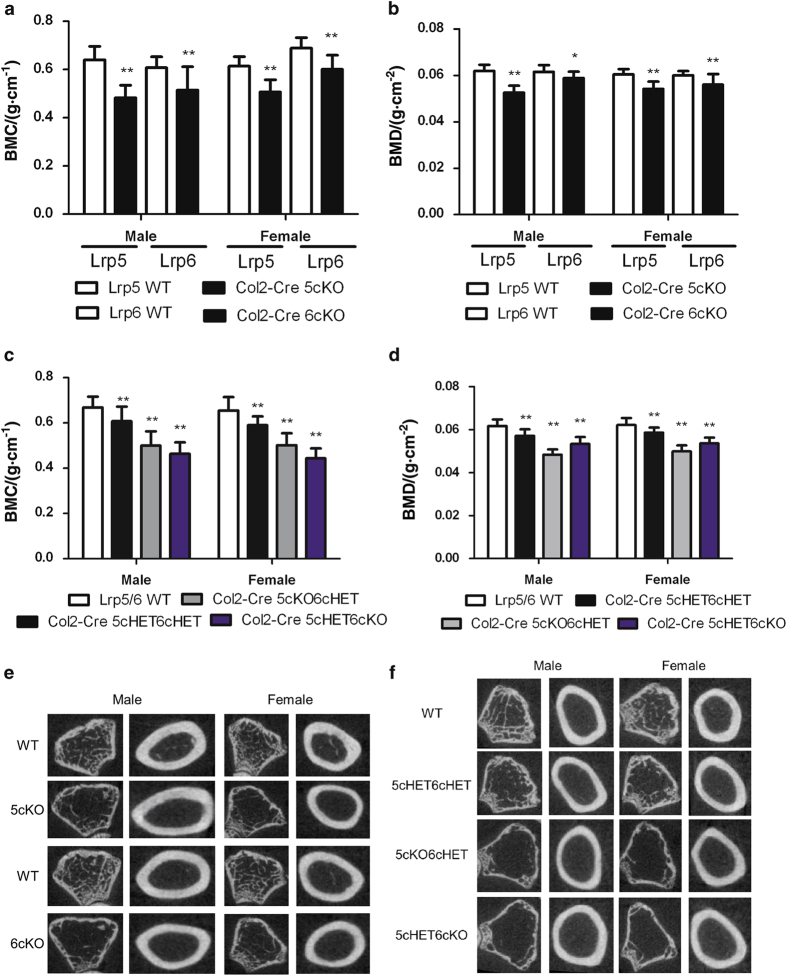
Adult *Col2-cre;Lrp5*^*Fl/FL*^ (5cKO) or *Col2-cre;Lrp6*^*Fl/FL*^ (6cKO) mice and mice with combinatorial deletions of *Lrp5* and *Lrp6* have low bone mass at six months of age. (**a**) Whole-body bone mineral content DEXA measurements (mean±s.d.) for male and female mice with single Lrp5 and single Lrp6 conditional loss of function mutations driven by *Col2-cre* and respective WT littermates; **P<0.01 compared to WT littermates. (**b**) Whole-body bone mineral density DEXA measurements (mean±s.d.) for male and female mice with single Lrp5 and single Lrp6 conditional loss of function mutations driven by *Col2-cre* and respective WT littermates; **P<0.01 and *P=0.01 compared to WT littermates. (**c**) Whole-body bone mineral content DEXA measurements (mean±s.d.) for male and female mice with double combinations of Lrp5 and Lrp6 conditional knockout or heterozygous loss of function mutation driven by *Col2-cre* and respective WT littermates; **P<0.01 compared with WT littermates. (**d**) Whole-body bone mineral density DEXA measurements (mean±s.d.) for male and female mice with combinations of Lrp5 and Lrp6 conditional knockout or heterozygous loss of function mutation driven by *Col2-cre* and respective WT littermates; **P<0.01 compared with WT littermates. (**e**) MicroCT cross sectional images of trabecular and cortical bone from male and female mice with single Lrp5 and single Lrp6 conditional knockout mutation driven by *Col2-cre* and of respective WT littermates. (**f**) MicroCT cross sectional images of trabecular and cortical bone from male and female mice with combinations of Lrp5 and Lrp6 conditional homozygous knockout or heterozygous loss of function mutations and of WT littermates. BMC, bone mineral content; BMD, bone mineral density.

To assess the effect of *Col2-cre*-induced loss of Lrp5 or Lrp6 on adult bone at 6 months of age, dual X-ray absorbtiometry was carried out to measure total body BMD. This demonstrated that BMD was significantly lower for male and female *Col2-cre;Lrp5*^*Fl/FL*^ and *Col2-cre;Lrp6*^*Fl/FL*^ mice relative to control littermates (Figure 5). Total body BMD was also significantly reduced in male and female *Col2-cre;Lrp5*^*Fl/+*^*;Lrp6*^*Fl/+*^, *Col2-cre;Lrp5*^*Fl/FL*^*;Lrp6*^*Fl/+*^, and *Col2-cre;Lrp5*^*Fl/+*^*;Lrp6*^*Fl/FL*^ mice compared with WT littermates (Figure 5).

A qualitative reduction in trabecular number and bone volume fraction, and an increase in trabecular spacing were observed in the distal femur of *Col2-cre;Lrp5*^*Fl/FL*^, *Col2-cre;Lrp6*^*Fl/FL*^, *Col2-cre;Lrp5*^*Fl/+*^*;Lrp6*^*Fl/+*^, *Col2-cre;Lrp5*^*Fl/FL*^*;Lrp6*^*Fl/+*^, and *Col2-cre;Lrp5*^*Fl/+*^*;Lrp6*^*Fl/FL*^ mice compared with WT littermates (Figure 5). Compared with respective WT littermates cortical cross sectional area and cortical thickness was significantly lower for male and female *Col2-cre;Lrp5*^*Fl/FL*^ and *Col2-cre;Lrp6*^*Fl/FL*^ (Table 3). Cortical mineral density was significantly lower for female *Col2-cre;Lrp5*^*Fl/FL*^ and *Col2-cre;Lrp6*^*Fl/FL*^ mice compared with WT (Table 3). Cortical thickness and mineral density were significantly lower for male and female mice with mutations in both Lrp5 and Lrp6 (Table 3). Cortical cross sectional area was significantly lower for all female mice with mutations in both Lrp5 and Lrp6 and male *Col2-cre;Lrp5*^*Fl/FL*^*;Lrp6*^*Fl/+*^ mice compared with respective WT mice (Table 3). Compared with respective WT littermates, trabecular bone volume fraction and number were significantly lower and separation significantly higher for *Col2-cre;Lrp5*^*Fl/FL*^ and *Col2-cre;Lrp6*^*Fl/FL*^ male and female mice (Table 3). Trabecular thickness was significantly lower for male *Col2-cre;Lrp5*^*Fl/FL*^ mice compared with WT (Table 3). Compared with respective WT littermates, trabecular bone volume fraction and number were significantly lower and separation higher for male and female *Col2-cre;Lrp5*^*Fl/FL*^*;Lrp6*^*Fl/+*^ and *Col2-cre;Lrp5*^*Fl/+*^*;Lrp6*^*Fl/FL*^ mice (Table 3). Trabecular thickness was significantly lower in female *Col2-cre;Lrp5*^*Fl/FL*^*;Lrp6*^*Fl/+*^ and *Col2-cre;Lrp5*^*Fl/+*^*;Lrp6*^*Fl/FL*^ mice and male *Col2-cre;Lrp5*^*Fl/FL*^*;Lrp6*^*Fl/+*^ mice compared with WT (Table 3).

### Adult mice with Col2-cre single Lrp5 or Lrp6 loss of function and double combinations of heterozygous and complete loss of Lrp5/6 function have normal articular cartilage and subchondral bone in knee joints

**Figure 6:**
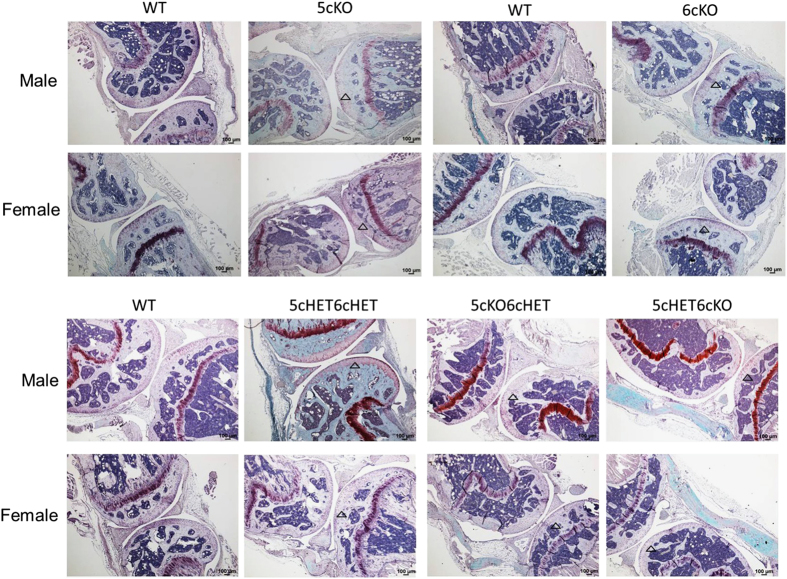
Adult mice carrying *Col2-cre*-mediated deletions of Lrp5 and/or Lrp6 display normal knee joints Safranin O/fast green stained sagittal sections of the articular cartilage of knee joints from male and female *Col2-cre*5cKO, *Col2-cre*6cKO, *Col2-cre*5cHET6cHET, *Col2-cre* 5cKO6cHET, *Col2-cre*5cHET6cKO, and wild-type littermate mice at 6 months of age (4×).

**Figure 7:**
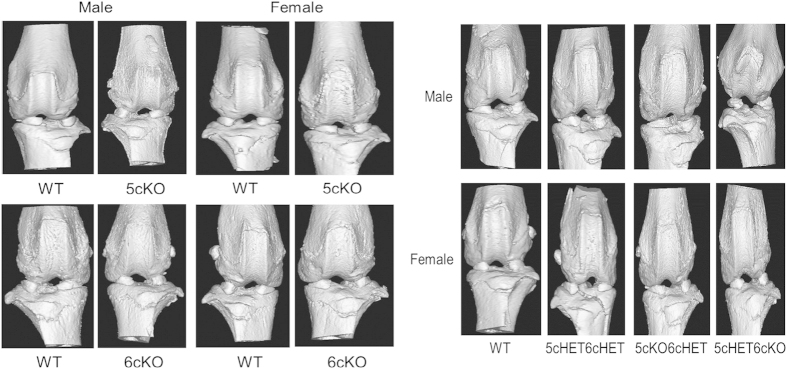
Adult mice carrying *Col2-cre*-mediated deletions of Lrp5 and/or Lrp6 have normal knee joints after evaluation by microCT. (Left) 3D reconstructed micro CT images of the knee joints from male and female *Col2-cre*5cKO, *Col2-cre*6cKO, and WT littermate mice. (Right) 3D reconstructed micro CT images of the knee joints from male and female *Col2-cre*5cHET6cHET, *Col2-cre* 5cKO6cHET, *Col2-cre*5cHET6cKO and WT littermate mice. WT, wild type.

There were no gross morphological differences in knee joints between WT and mutant mice. Safranin O staining intensity was slightly reduced in articular cartilage from *Col2-cre;Lrp5*^*Fl/FL*^*;Lrp6*^*Fl/+*^ and *Col2-cre;Lrp5*^*Fl/+*^*;Lrp6*^*Fl/FL*^ mice compared with littermates (Figure 6). The tidemark was well preserved in all mice and there was no obvious difference in cartilage thickness (Figure 6). Upon microcomputed tomography (microCT), knee joint subchondral bone morphology also appeared similar between mutant mice and WT littermates and there was no pathological evidence of degenerative joint diseases including subchondral bone erosion or osteophyte formation (Figure 7).

## Discussion

Studies have linked Wnt/β-catenin signaling to degenerative joint disease in humans and mice.^19,23,38–43^ The fact that *Col2-cre*-mediated deletion of *Lrp5* results in normal appearing knee joints is not surprising given that global deletion of *Lrp5* was previously shown to not alter skeletal morphogenesis.^25,44–46^ However, homozygosity for an inactivating allele of *Lrp6* results in neonatal lethality,^24^ so it was previously not possible to evaluate the effects of loss of Lrp6 on the adult knee. *Col2-cre*-mediated deletion of Lrp6 did not result in any discernable knee phenotype. Furthermore, mice in which either Lrp5 or Lrp6 were homozygously deleted with *Col2-cre*, whereas the other gene was heterozygously deleted also displayed normal knee morphology. As homozygous deletion of both genes results in embryonic lethality,^26^ it is not possible to assess the effects of double deletion on the adult knee in mice carrying global deletions. We feel this is consistent with the idea that it is likely that retention of at least one allele of either Lrp5 or Lrp6 within the descendants of *Col2-cre* expressing cells is sufficient to mediate normal joint development. Alternatively, it is also possible that signaling from either Lrp5 or Lrp6 is not required for normal joint development. The use of chondrocyte-specific cre drivers that can be controlled temporally to induce simultaneous loss of both genes may be a way to fully discern between these two possibilities.

The presence of decreased bone mass in mice carrying deletions in Lrp5 and Lrp6 is consistent with previous observations that mutations in one of both of these genes within the osteochondral lineage cause reductions in bone mass.^1^^,^^25^^,^^31^^,^^35^^,^^45^^,^^47^ We did not carry out detailed histomorphometry in the context of these studies, but previously work showed that mice carrying OCN-cre-mediated deletions of either *Lrp5* or *Lrp6* developed osteopenia^35^ and therefore speculate that this could be the underlying mechanism for the reduced bone mass observed.

An alternative model in which Lrp5 functions to control bone mass via regulating the production of serotonin from enterchromaffin cells of the cells of the duodenum has also been proposed.^48–50^ However, this latter model has been debated.^51–54^ In any event, reductions in bone mass caused by loss of Lrp5 and/or Lrp6 in the descendants of *Col2-cre*-expressing cells could be either owing to widespread leakiness of the *Col2-cre* transgene within osteoblasts or reflect significant contribution of *Col2-cre* expressing cells to the osteoblast lineage found in the mature skeleton.

Although a detailed assessment of body composition was not part of this study, we did not observe differences in body weight that might be of interest for future studies. Assessment of the mice at the time of DEXA analysis demonstrated that both male (12% decrease) and female (9% decrease) *Col2-cre;Lrp5*^*Fl/FL*^ mice weighed significantly less than their WT counterparts. *Col2-cre;Lrp6*^*Fl/FL*^ female mice also weighed significantly less than controls (17% reduction), whereas male mice displayed a downward trend (4% decrease) that did not reach statistical significance. Whether this is related to relative changes in lean body mass and/or energy expenditure^55^ could be assessed in future studies.

The observation that Lrp5 and Lrp6 act in an overlapping and/or redundant manner within the descendants of *Col2-cre* expressing cells is consistent with observations in a wide variety of other cell types and tissues. These include mice carrying global deletions in the two genes as well as mice in which both genes are deleted in cells derived from the following cre drivers: *Osteocalcin-cre* (mature osteoblasts and osteocytes),^35^ *Dermo1-cre* (osteochondral progenitors),^31^ and *Albumin-cre* (hepatocytes).^56^ Future work will evaluate how Lrp5 and Lrp6 coordinately regulate downstream signaling pathways from Wnt ligands^32^^,^^57,58^ that are necessary for normal development in skeletal tissues.

## Figures and Tables

**Table 1:** Mouse strains used in this work

*Nomenclature*	*Extended allele name*	*JAX database stock #*	*Genetic background*	*References*
*Col2-cre*	Tg(Col2a1cre)1Bhr/J	003554	C57Bl/6 J; SJL	^29^
Lrp5flox	*Lrp5*^*tm1.1Vari*^/J	026269	129/SvJ; C57Bl/6 J	^31^
Lrp6flox	*Lrp6*^*tm1.1Vari*^/J	026267	129/SvJ; C57Bl/6 J	^31^
Mt/mG	*Gt(ROSA)26Sor*^*tm4(ACTB-tdTomato,-EGFP)Luo*^/J	007576	129X1/SvJ	^33^

WT, wild type.

In each case, the nomenclature used in this study is listed, followed by the strain name and stock number for each strain at the Jackson Laboratories (JAX).

**Table 2:** Summary of nomenclature for genetically engineered mice characterized in this study

*Nomenclature*	*Abbreviation in figures*	*Col2-cre genotype*	*Lrp5*^*Flox*^*genotype*	*Lrp6*^*Flox*^*genotype*	*Survival*
Control	WT	+/+	All	All	Viable
*Col2-cre*;Lrp5^Fl/FL^	5cKO	TG/+	Flox/Flox	+/+	Viable
*Col2-cre*;Lrp6^Fl/FL^	6cKO	TG/+	+/+	Flox/Flox	Viable
*Col2-cre*;Lrp5^Fl/+^;Lrp6^Fl/+^	5cHET6cHET	TG/+	Flox/+	Flox/+	Viable
*Col2-cre*;Lrp5^Fl/FL^;Lrp6^Fl/+^	5cKO6cHET	TG/+	Flox/Flox	Flox/+	Viable
*Col2-cre*;Lrp5^Fl/+^;Lrp6^Fl/FL^	5cHET6cKO	TG/+	Flox/+	Flox/Flox	Viable
*Col2-cre*;Lrp5^Fl/FL^;Lrp6^Fl/FL^	5cKO6cKO	TG/+	Flox/Flox	Flox/Flox	Neonatal lethal

WT, wild type.

A “+” is indicative of an unmodified (wild type) locus. A designation of “TG” denotes that a mouse is carrying the *Col2-cre* transgene while “Flox” indicates the presence of an allele of a gene that can be inactivated upon exposure to cre recombinase. The abbreviations used in the figures to refer to the different genotypes is also shown.

**Table 3:** Average microCT cortical and trabecular bone geometrical measurements on samples from mice at 6 months of age

	Lrp5				Lrp6				Lrp5Lrp6							
		Male		Female		Male		Female		Male				Female		
Bone	WT 10	cKO 10	WT 10	cKO 10	WT 10	cKO 10	WT 10	cKO 10	WT 10	cHETcHET 10	cKOcHET 9	cHETcKO 6	WT 10	cHETcHET 10	cKOcHET 12	cHETcKO 10
*Trabecular bone*																
Percent bone volume/%	**20.114**	**6.447**	**13.676**	**8.212**	**15.602**	**7.024**	**9.561**	**5.506**	**12.762**	**10.362**	**2.206**	**3.945**	**12.050**	**7.049**	**4.385**	**4.880**
Standard deviation	4.345	3.552	3.204	2.099	6.054	3.918	2.717	1.981	4.268	5.338	0.545	1.579	2.067	1.814	2.751	1.646
*P* value		1.1E−06**		6.14E−04**	**0.032**	0.004**		0.002**		**0.307**	3.56E−05**	1.05E−04**		3.72E−05**	7.16E−07**	2.71E−07**
Trabecular thickness/mm	**0.033**	**0.026**	**0.029**	**0.027**	**0.032**	**0.028**	**0.030**	**0.029**	**0.031**	**0.032**	**0.026**	**0.030**	**0.034**	**0.032**	**0.027**	**0.028**
Standard deviation	0.003	0.004	0.003	0.002	0.004	0.004	0.001	0.003	0.005	0.006	0.002	0.005	0.003	0.003	0.003	0.002
*P* value		2.54E−04**		**0.180**		**0.075**		**0.158**		**0.678**	0.008**	**0.791**		**0.228**	7.34E−05**	9.12E−05**
Trabecular separation/mm	**0.136**	**0.452**	**0.192**	**0.326**	**0.190**	**0.448**	**0.312**	**0.552**	**0.236**	**0.342**	**1.197**	**0.834**	**0.254**	**0.452**	**0.986**	**0.601**
Standard deviation	0.023	0.170	0.048	0.085	0.051	0.169	0.086	0.178	0.080	0.145	0.288	0.280	0.045	0.114	0.860	0.201
*P* value		3.29E−04**		0.001**		0.001**		0.003**		**0.075**	7.11E−06**	0.004**		4.30E−04**	0.017*	5.16E−04**
Trabecular number/mm^−1^	**5.992**	**2.374**	**4.671**	**2.983**	**4.780**	**2.408**	**3.137**	**1.887**	**4.063**	**3.040**	**0.860**	**1.266**	**3.537**	**2.165**	**1.553**	**1.788**
Standard deviation	0.782	0.919	0.735	0.670	1.182	0.940	0.885	0.582	1.110	1.023	0.183	0.357	0.437	0.461	0.894	0.675
*P* value		5.5E−08**		7.83E−05**		0.000**		0.003**		**0.057**	9.06E−06**	1.61E−05**		4.36E−06**	6.34E−06**	8.39E−06**
																
*Cortical bone*																
Mean total crossectional bone area/mm^2^	**0.988**	**0.726**	**0.920**	**0.720**	**0.955**	**0.826**	**0.861**	**0.713**	**0.913**	**0.882**	**0.646**	**0.776**	**0.944**	**0.842**	**0.629**	**0.676**
Standard deviation	0.076	0.121	0.057	0.071	0.066	0.080	0.052	0.083	0.085	0.112	0.077	0.141	0.060	0.054	0.080	0.041
*P* value		5.86E−05**		4.33E−06**		0.002**		4.04E−04**		**0.512**	3.24E−06**	**0.089**		0.001**	3.42E−09**	7.92E−09**
Crossectional thickness/mm	**0.229**	**0.191**	**0.230**	**0.193**	**0.210**	**0.201**	**0.212**	**0.195**	**0.205**	**0.189**	**0.161**	**0.188**	**0.225**	**0.208**	**0.168**	**0.183**
Standard deviation	0.009	0.017	0.007	0.010	0.010	0.009	0.007	0.012	0.010	0.009	0.010	0.015	0.012	0.008	0.017	0.007
*P* value		3.18E−05**		5.27E−08**		**0.071**		0.002**		0.002**	5.52E−08**	0.050*		0.003**	3.36E−08**	2.61E−07**
Mineral density/(g·cm^−2^)	1.151	1.122	**1.160**	**1.124**	**1.109**	**1.107**	**1.135**	**1.119**	**1.170**	**1.087**	**1.078**	**1.103**	**1.189**	**1.136**	**1.087**	**1.103**
Standard deviation	0.038	0.037	0.021	0.020	0.030	0.027	0.013	0.018	0.040	0.034	0.029	0.043	0.045	0.020	0.065	0.015
*P* value		**0.117**		0.001**		**0.926**		0.052*		1.77E−04**	4.48E−05**	0.017*		0.007**	5.32E−04**	2.24E−04**

Abbreviation: WT, wild type.

*P*-values were calculated with respect to WT littermates; *P<0.05 and **P<0.01.
